# Anti-Inflammatory Effects of *Angelica sinensis* (Oliv.) Diels Water Extract on RAW 264.7 Induced with Lipopolysaccharide

**DOI:** 10.3390/nu10050647

**Published:** 2018-05-21

**Authors:** Young-Jin Kim, Ji Young Lee, Hyun-Ju Kim, Do-Hoon Kim, Tae Hee Lee, Mi Suk Kang, Wansu Park

**Affiliations:** College of Korean Medicine, Gachon University, Seong-nam 13120, Korea; godsentry@naver.com (Y.-J.K.); oxygen1119@naver.com (J.Y.L.); eternity0304@daum.net (H.-J.K.); chulian@gachon.ac.kr (D.-H.K.); ophm5418@gachon.ac.kr (T.H.L.); cyberdoc@gachon.ac.kr (M.S.K.)

**Keywords:** *Angelica sinensis*, anti-inflammatory, macrophage, cytokine, calcium, nitric oxide, JAK-STAT

## Abstract

The dry root of *Angelica sinensis* (Oliv.) Diels, also known as “female ginseng”, is a popular herbal drug amongst women, used to treat a variety of health issues and cardiovascular diseases. The aim of this study is to evaluate the detailed molecular mechanism for anti-inflammatory effects of *Angelica sinensis* root water extract (ASW). The anti-inflammatory effect of ASW on lipopolysaccharide (LPS)-induced RAW 264.7 mouse macrophages was evaluated by the tetrazolium-based colorimetric assay (MTT), Griess reagent assay, multiplex cytokine assay, real time reverse transcription polymerase chain reaction (RT-PCR), and Fluo-4 calcium assay. ASW restored cell viability in RAW 264.7 at concentrations of up to 200 µg/mL. ASW showed notable anti-inflammatory effects. ASW exhibited IC_50_ = 954.3, 387.3, 191.7, 317.8, 1267.0, 347.0, 110.1, 573.6, 1171.0, 732.6, 980.8, 125.0, and 257.0 µg/mL for interleukin (IL)-6, tumor necrosis factor (TNF)-α, monocyte chemotactic activating factor (MCP)-1, regulated on activation, normal T cell expressed and secreted (RANTES), granulocyte colony-stimulating factor (G-CSF), granulocyte macrophage colony-stimulating factor (GM-CSF), vascular endothelial growth factor (VEGF), lipopolysaccharide-induced CXC chemokine (LIX), macrophage inflammatory protein (MIP)-1α, MIP-1β, MIP-2, IL-10, and intracellular calcium, respectively. Additionally, ASW inhibited the LPS-induced production of nitric oxide and the LPS-induced mRNA expression of CHOP (GADD153), Janus kinase 2 (JAK2), signal transducers and activators of transcription 1 (STAT1), first apoptosis signal receptor (FAS), and c-Fos, NOS2, and PTGS2 (COX2) in RAW 264.7 significantly (*p* < 0.05). Data suggest that ASW exerts an anti-inflammatory effect on LPS-induced RAW 264.7 via NO-bursting/calcium-mediated JAK-STAT pathway.

## 1. Introduction

Immunity is a core function of human life against pathologic infections. It is our first-defense mechanism against infection as an innate immune activity. Innate immune activity includes inflammasome and pyroptosis [1]. Inflammasome (pyroptosome) provokes cytokine production and leads to programmed cell death-pyroptosis. Pyroptosis, distinct from apoptosis, is a programmed, immune cell death caused by intracellular pathogens [1]. Inflammation is indispensable during an immune reaction against pathogens, such as bacteria, viruses, and fungi. With inflammation, we experience heat, pain, edema, and redness. Consequently, inflammatory mediators can be produced massively against infectious pathogens, which may be hazardous to the human host. Thus, it is important to both remove pathogens, and modulate hyper-inflammatory activity and excessive production of inflammatory mediators, such as nitric oxide (NO) and cytokines.

NO, which can act as a free radical, is not only a strong toxic molecule to combat invasive pathogens (bacteria, virus, etc.), but also a powerful vasodilator in the cellular signaling pathway of inflammation. NO is regarded as playing a major role in the fatal hypotension of septic shock and sepsis.

Cytokines, including chemokines and growth factors, also play an important role in hypersensitive reactions against both exogenous and endogenous antigens. Cytokines may be maturated by an inflammasome-promoted process.

In 2011, Chao et al. reported that *Angelica sinensis* (Oliv.) Diels ethyl acetate extract significantly inhibited NF-kappaB trans-activation activity with down-regulating NO and cytokines from lipopolysaccharide (LPS), plus IFN-γ-induced RAW 264.7 [2]. However, the effect of *Angelica sinensis* root water extract (ASW) on LPS-induced macrophages to our knowledge has not been reported.

In the present study, we investigated the anti-inflammatory effects of ASW using multiplex cytokine assay and quantitative real time reverse transcription polymerase chain reaction (RT-PCR) in lipopolysaccharide (LPS)-induced RAW 264.7 mouse macrophages. The resulting data suggests that ASW exerts anti-inflammatory effects via NO-bursting/calcium-mediated Janus kinase (JAK)-signal transducers and activators of transcription (STAT) pathway.

## 2. Materials and Methods

### 2.1. Reagents

Dulbecco’s modified Eagle’s medium (DMEM) was purchased from Gibco BRL (Grand Island, NY, USA) and all other chemicals for the cell culture were obtained from Merck Millipore (Darmstadt, Germany).

### 2.2. Preparation of ASW

The commercial product of *Angelica sinensis* root was obtained from Omniherb (Daegu, Korea) and authenticated by Professor Y. J. Lee of Gachon University in June 2015. A voucher specimen (No. 2015-0027) was deposited at the Department of Pathology in Gachon University College of Korean Medicine. Because herbal drugs have been traditionally extracted using water, in the present study, *Angelica sinensis* root was extracted with boiling water for 2 h, filtered, and then lyophilized (yield: 50.63%). The powdered extract was dissolved in saline and then filtered through a 0.22 µm syringe filter.

### 2.3. Total Flavonoid Content

The total flavonoid content of ASW was determined using the diethylene glycol colorimetric method. Briefly, the sample solution (20 µL of 2 mg/mL ASW) was mixed with 200 µL of diethylene glycol and 20 µL of 1 N NaOH. The sample absorbance was read at 405 nm after 1 h incubation at 37 °C. Rutin was used as a reference standard and the total flavonoid content was expressed as milligrams of rutin equivalents (mg RE/g extract) [3].

### 2.4. Cell Viability

RAW 264.7 were obtained from Korea Cell Line Bank (Seoul, Korea). RAW 264.7 (passage number 7) were cultured with DMEM supplemented with 10% FBS containing 100 U/mL of penicillin and 100 µg/mL of streptomycin at 37 °C in a 5% CO_2_ humidified incubator according to the protocol of previous study [4]. Before experimental assays, RAW 264.7 were washed with phosphate buffer saline. Briefly, RAW 264.7 were cultured with samples for 24 h in 96-well plates (each well containing 10,000 cells) in order to verify the toxicity of ASW. After 24 h culture with samples, cell viability was confirmed with the tetrazolium-based colorimetric assay (MTT) according to the protocol of a previous study [4].

### 2.5. NO Concentration

After 24-h incubation with samples in 96-well plates, NO levels in each well containing 10,000 cells were identified using the Griess reagent according to a previous study [4].

### 2.6. Multiplex Cytokine Assay

After 24-h incubation with samples in 96-well plates, the following cytokines production from each well containing 10,000 cells was analyzed: interleukin (IL)-6; IL-10, tumor necrosis factor (TNF)-α; granulocyte colony-stimulating factor (G-CSF); granulocyte macrophage colony-stimulating factor (GM-CSF); lipopolysaccharide-induced CXC chemokine (LIX; CXCL5); monocyte chemotactic activating factor (MCP)-1; macrophage inflammatory protein (MIP)-1α, MIP-1β, MIP-2, RANTES (CCL5; regulated on activation, normal T cell expressed and secreted), vascular endothelial growth factor (VEGF); interferon gamma-induced protein 10 (IP-10; CXCL10); and leukemia inhibitory factor (LIF). Cytokines were measured using a Luminex assay based on xMAP technology with multiplex cytokine assay kits and a Bio-Plex 200 suspension array system (Bio-Rad, Hercules, CA, USA), as described previously [4,5,6,7,8,9]. The assay used in this experiment was designed for the multiplexed quantitative measurement of multiple cytokines in a single well, using as little as 50 µL of cell culture supernatant. Baicalein, a well-known anti-inflammatory flavonoid, was used as a positive control.

### 2.7. Intracellular Calcium Assay

Fluo-4 AM is a well-known fluorescent Ca^2+^ indicator used for in-cell measurement of calcium signaling. After 18 h treatment with samples in 96-well plates, the intracellular signaling from each well containing 100,000 cells was identified using Fluo-4 NW Calcium Assay Kits (Thermo Fisher Scientific, Waltham, MA, USA) according the protocol of our previous study [5,6,7]. The assay was done with a spectrofluorometer (Dynex, West Sussex, UK) with excitation and emission filters of 485 nm and 535 nm, respectively. Indomethacin, which is known to impair mitochondrial calcium uptake, was used as a positive control.

### 2.8. Real Time RT-PCR Assay

After 18-h incubation with samples in 6-well plates, the mRNA expression of STAT1, STAT3, C/EBP homologous protein (CHOP; GADD153; DDIT3), JAK2, first apoptosis signal receptor (FAS), c-Fos, nitric oxide synthase 2 (NOS2), and prostaglandin-endoperoxide synthase 2 (PTGS2; COX2) in each well containing 300,000 cells were evaluated with real time RT-PCR using Experion Automatic Electrophoresis System (Bio-Rad) and Bio-Rad CFX 96 Real-Time PCR Detection System (Bio-Rad) [10,11]. Cells were lysed and Total RNA was isolated using the NucleoSpin RNA kit (Macherey-Nagel, Duren, Germany) according to the manufacturer’s instructions. The β-Actin was used as a reference. Primers used are listed in Table 1.

### 2.9. Statistical Analysis

Experimental results are presented as mean ± standard deviation. Experiments were done more than three times. Data analysis was performed by Student *t*-test or one-way analysis of variance test followed by Tukey’s multiple comparison test, as appropriate. For statistics, the program Graphpad Prism 4.01 (Graphpad Software, La Jolla, CA, USA) was used. A *p*-value < 0.05 was considered statistically.

## 3. Results

### 3.1. Determination of the Total Flavonoid Content of ASW

Our data represented that the total flavonoid content of ASW was 5.14 mg RE/g extract.

### 3.2. Effects of ASW on Cell Viability

**Figure 1:**
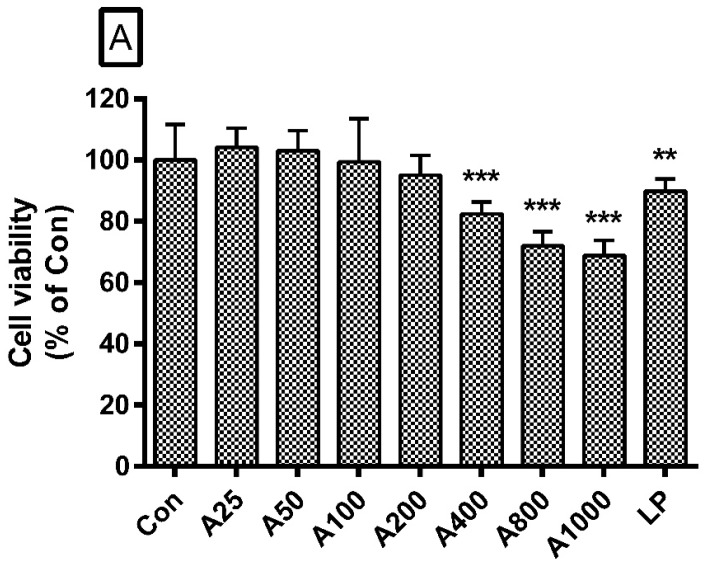

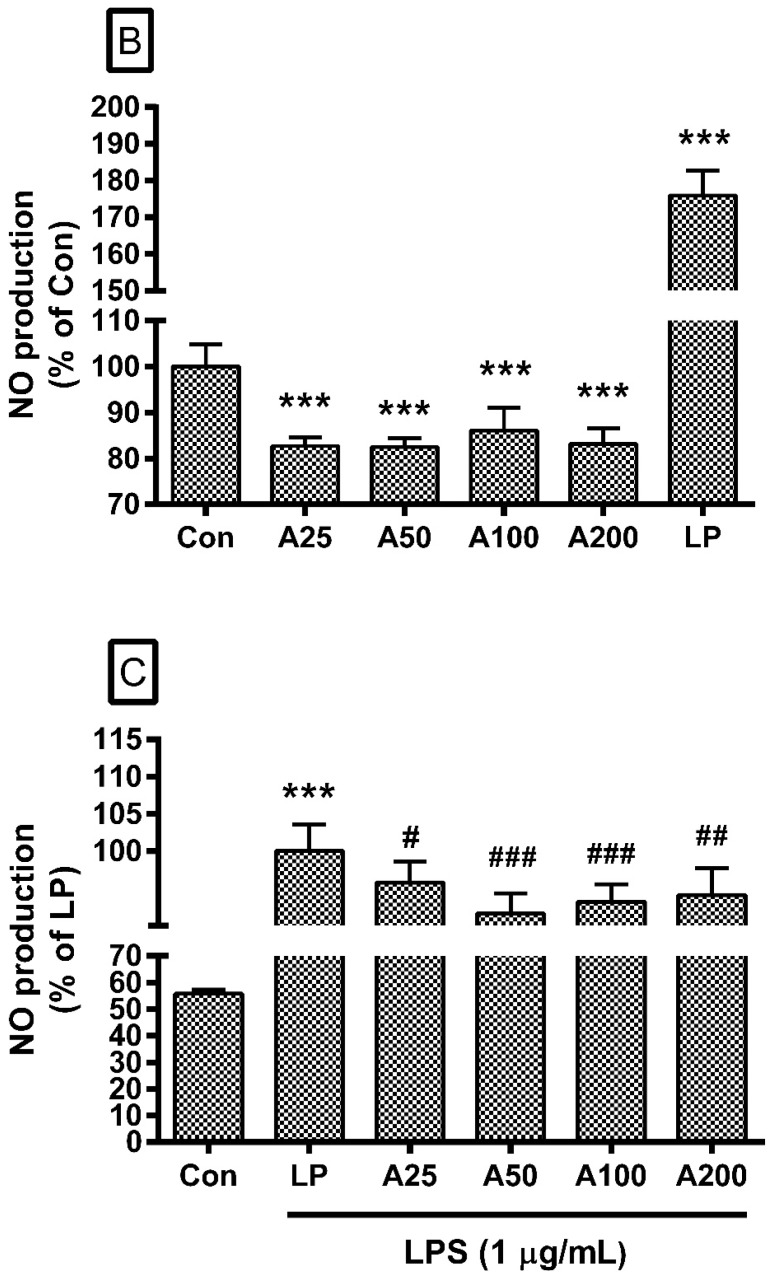
Effects of *Angelica sinensis* root water extract (ASW) on cell viability (**A**) and nitric oxide (NO) production (**B**,**C**). Cells were treated for 24 h. Con means the group treated with media only. LP means the group treated with 1 µg/mL of lipopolysaccharide (LPS) alone. A25, A50, A100, A200, A400, A800, and A1000 mean 25, 50, 100, 200, 400, 800, and 1000 µg/mL of ASW respectively. Values are the mean ± standard deviation of at least three independent experiments. Statistical significance was calculated by Student *t*-test (**A**,**B**) and one-way ANOVA and a Tukey multiple comparison test (**C**). ** *p* < 0.01 vs. Con; *** *p* < 0.001 vs. Con; ^#^ *p* < 0.05 vs. LP; ^##^ *p* < 0.01 vs. LP; ^###^ *p* < 0.001 vs. LP.

In this study, LPS (1 µg/mL) caused a significant reduction in cell viability when compared with the negative control; ASW at concentrations of 400, 800, and 1000 µg/mL caused a significant reduction in cell viability as well (*p* < 0.05). However, ASW at concentrations of 25, 50, 100, and 200 µg/mL did not show any cytotoxic activity in this assay. In detail, the cell viability in RAW 264.7 cells which were incubated with ASW at concentrations of 25, 50, 100, 200, 400, 800, and 1000 µg/mL for 24 h were 104.16 ± 6.32%, 103.1 ± 6.58%, 99.5 ± 14.03%, 95.01 ± 6.58%, 82.49 ± 3.85%, 72.1 ± 4.58%, and 68.86 ± 4.84% of the control group treated with media only, respectively. With these results, ASW concentrations of up to 200 µg/mL were chosen for subsequent experiments (Figure 1A).

### 3.3. Effects of ASW on NO Production

In this study, LPS caused a significant increase in NO production when compared with the negative control, but ASW at concentrations of 25, 50, 100, and 200 µg/mL caused a significant reduction in NO production when compared with the negative control (*p* < 0.05). In detail, the production of NO in RAW 264.7 incubated with ASW at concentrations of 25, 50, 100, and 200 µg/mL for 24 h were 82.73 ± 1.9%, 82.52 ± 1.87%, 86.14 ± 4.93%, and 83.16 ± 3.42% of the control group treated with media only, respectively (Figure 1B). In addition, ASW significantly inhibited excessive production of NO in LPS-induced RAW 264.7 (Figure 1C) (*p* < 0.05). The production of NO in LPS-induced RAW 264.7 incubated with ASW at concentrations of 25, 50, 100, and 200 µg/mL for 24 h were 95.78 ± 2.86%, 91.65 ± 2.71%, 93.23 ± 2.34%, and 94.11 ± 3.64% of the group treated with LPS only, respectively.

### 3.4. Effect of ASW on Cytokine Production

**Figure 2:**
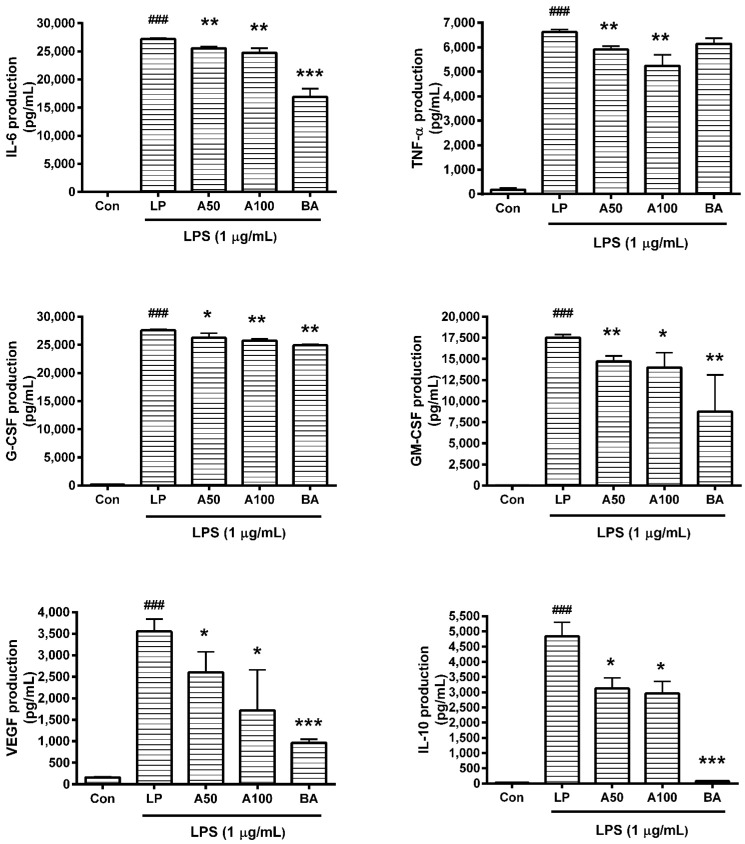
Effects of *Angelica sinensis* root water extract (ASW) on interleukin (IL)-6, tumor necrosis factor (TNF)-α, granulocyte colony-stimulating factor (G-CSF), granulocyte macrophage colony-stimulating factor (GM-CSF), vascular endothelial growth factor (VEGF), and IL-10 production in lipopolysaccharide (LPS)-induced RAW 264.7. Cells were treated for 24 h. Con means the group treated with media only. LP means the group treated with 1 µg/mL of LPS alone. A50 and A100 mean 50 and 100 µg/mL of ASW, respectively. BA means baicalein (100 µM). Values are the mean ± standard deviation of at least three independent experiments. Statistical significance was calculated by one-way ANOVA and a Tukey multiple comparison test. ^###^ *p* < 0.001 vs. Con; * *p* < 0.05 vs. LP; ** *p* < 0.01 vs. LP; *** *p* < 0.001 vs. LP.

**Figure 3:**
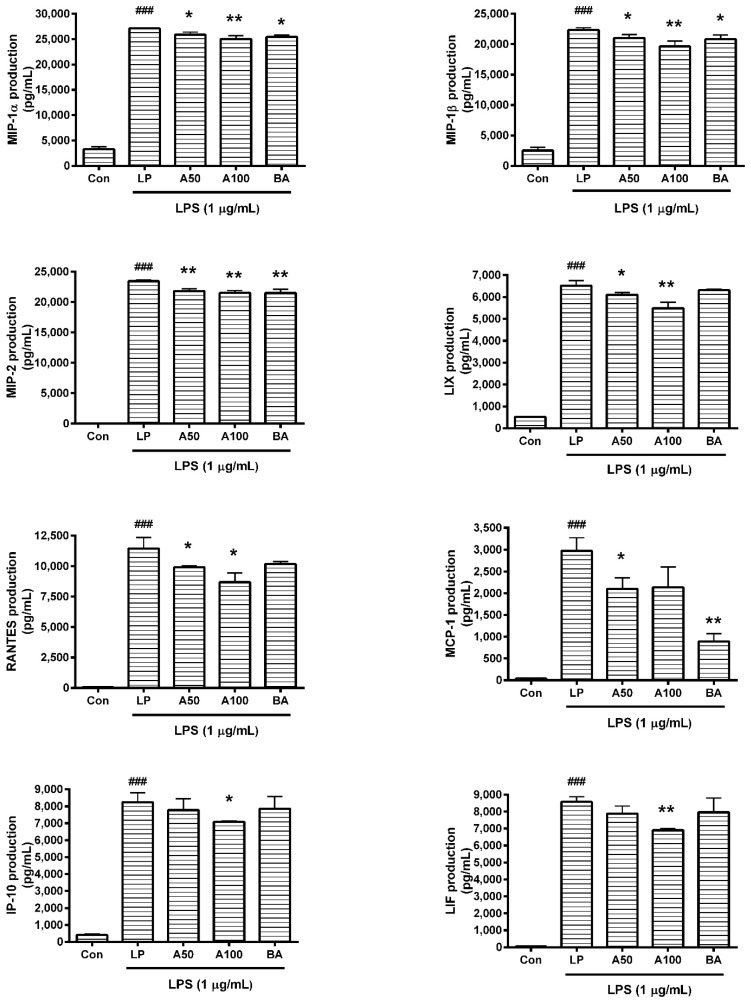
Effects of *Angelica sinensis* root water extract (ASW) on macrophage inflammatory protein (MIP)-1α, MIP-1β, MIP-2, lipopolysaccharide-induced CXC chemokine (LIX), regulated on activation, normal T cell expressed and secreted (RANTES), monocyte chemotactic activating factor (MCP)-1, interferon gamma-induced protein 10 (IP-10), and leukemia inhibitory factor (LIF) production in lipopolysaccharide (LPS)-induced RAW 264.7. Cells were treated for 24 h. Con means the group treated with media only. LP means the group treated with 1 µg/mL of LPS alone. A50 and A100 mean 50 and 100 µg/mL of ASW respectively. BA means baicalein (100 µM). Values are the mean ± standard deviation of at least three independent experiments. Statistical significance was calculated by one-way ANOVA and a Tukey multiple comparison test. ^###^ *p* < 0.001 vs. Con; * *p* < 0.05 vs. LP; ** *p* < 0.01 vs. LP.

In this assay, data showed that ASW decreased production of cytokines such as IL-6, IL-10, TNF-α, G-CSF, GM-CSF, VEGF, MIP-1α, MIP-1β, MIP-2, LIX, RANTES, and IP-10 in LPS-induced RAW 264.7 (Figure 2 and Figure 3) significantly (*p* < 0.05). The detailed results of this assay are as follows: IL-6 productions in LPS-induced RAW 264.7 incubated with ASW at concentrations of 50 and 100 µg/mL for 24 h were 93.96 ± 1.02% and 91.1 ± 2.89% of the LPS-treated group value, respectively; TNF-α productions were 89.14 ± 2.17% and 79.12 ± 6.8%; MCP-1 productions were 70.7 ± 8.7% and 71.99 ± 15.73%; RANTES productions were 86.66 ± 1.12% and 75.9 ± 6.75%; G-CSF productions were 95.17 ± 2.94% and 93.24 ± 1.27%; GM-CSF productions were 83.89 ± 3.68% and 79.86 ± 10.03%; VEGF productions were 73.38 ± 13.34% and 48.45 ± 26.46%; LIX productions were 93.62 ± 1.52% and 84.2 ± 4.09%; MIP-1α productions were 95.54 ± 1.77% and 92.33 ± 2.36%; MIP-1β productions were 93.98 ± 2.66% and 87.78 ± 4.02%; MIP-2 productions were 93.09 ± 1.53% and 91.88 ± 1.61%; IL-10 productions were 64.6 ± 7.29% and 61.19 ± 8.06%; IP-10 productions were 94.42 ± 8.05% and 86.12 ± 0.33%; LIF productions were 91.95 ± 5.17% and 80.51 ± 1.22%.

### 3.5. Effect of ASW on Intracellular Calcium Release

**Figure 4:**
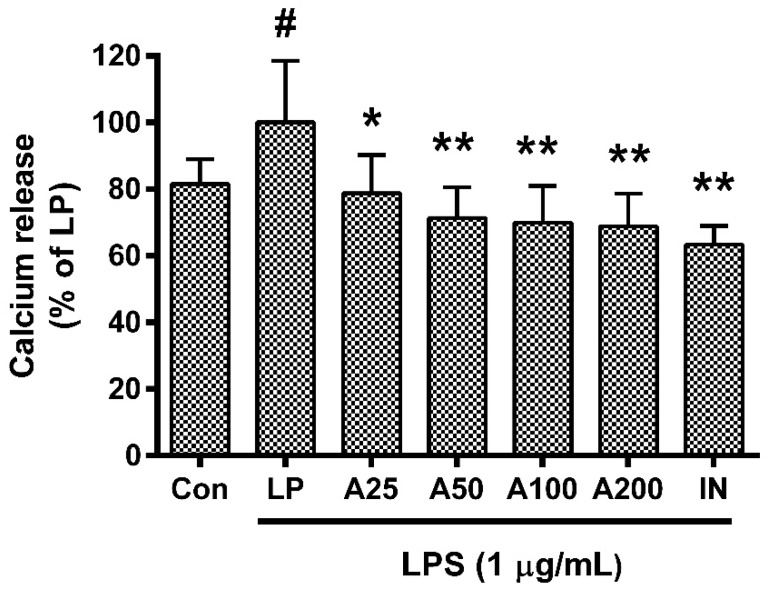
Effects of *Angelica sinensis* root water extract (ASW) on calcium release in lipopolysaccharide (LPS)-induced RAW 264.7. Cells were treated for 18 h. Con means the group treated with media only. LP means the group treated with 1 µg/mL of LPS alone. A25, A50, A100, and A200 mean 25, 50, 100, and 200 µg/mL of ASW respectively. IN means indomethacin (0.5 µM). Values are the mean ± standard deviation of at least three independent experiments. Statistical significance was calculated by one-way ANOVA and a Tukey multiple comparison test. ^#^ *p* < 0.05 vs. Con; * *p* < 0.05 vs. LP; ** *p* < 0.01 vs. LP.

ASW significantly inhibited the calcium release in LPS-induced RAW 264.7 (Figure 4) (*p* < 0.05). In detail, the calcium release in LPS-induced RAW 264.7 incubated with ASW at concentrations of 25, 50, 100, and 200 µg/mL for 18 h were 78.79 ± 11.42%, 71.33 ± 9.25%, 69.94 ± 10.99%, and 68.84 ± 9.8% of the group treated with LPS only, respectively. The IC_50_ value of ASW was 257 µg/mL.

### 3.6. Effect of ASW on mRNA Expression

**Figure 5:**
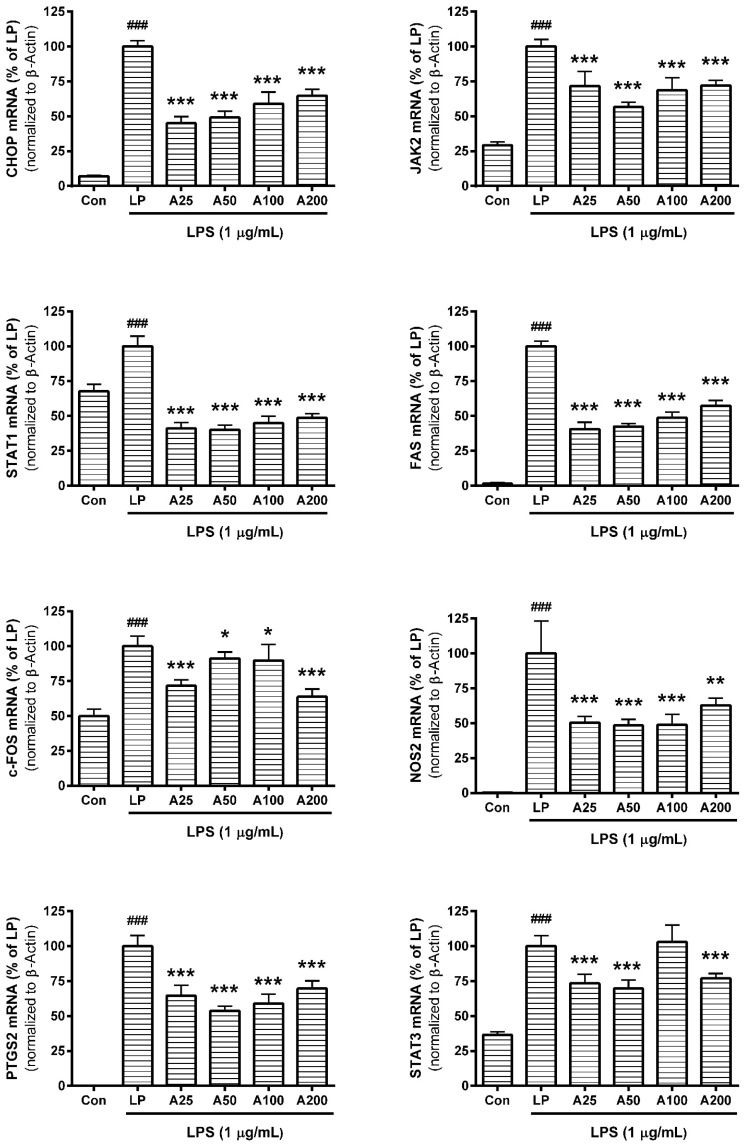
Effects of *Angelica sinensis* root water extract (ASW) on the mRNA expression of C/EBP homologous protein (CHOP), janus kinase 2 (JAK2), signal transducers and activators of transcription 1 (STAT1), first apoptosis signal receptor (FAS), c-Fos, nitric oxide synthase 2 (NOS2), prostaglandin-endoperoxide synthase 2 (PTGS2), and STAT3 in lipopolysaccharide (LPS)-induced RAW 264.7. Cells were treated for 18 h. Each mRNA was normalized to the housekeeping gene β-Actin mRNA. Con means the group treated with media only. LP means the group treated with 1 µg/mL of LPS alone. A25, A50, A100, and A200 mean 25, 50, 100, and 200 µg/mL of ASW respectively. Values are the mean ± standard deviation of at least three independent experiments. Statistical significance was calculated by one-way ANOVA and a Tukey multiple comparison test. ^###^ *p* < 0.001 vs. Con; * *p* < 0.05 vs. LP; ** *p* < 0.01 vs. LP; *** *p* < 0.001 vs. LP.

In this assay, data showed that ASW decreased mRNA expressions of CHOP, JAK2, STAT1, FAS, c-FOS, NOS2, and PTGS2 in LPS-induced RAW 264.7 significantly (Figure 5) (*p* < 0.05). The detailed results of this assay were as follows: the mRNA expression of CHOP in RAW 264.7 incubated with LPS plus ASW (25 µg/mL), LPS plus ASW (50 µg/mL), LPS plus ASW (100 µg/mL), and LPS plus ASW (200 µg/mL) for 18 h were 45.08 ± 4.69%, 49.1 ± 4.52%, 59.04 ± 8.32%, and 64.69 ± 4.63% of the group treated with LPS only, respectively; JAK2 were 45.08 ± 4.69%, 49.1 ± 4.52%, 59.04 ± 8.32%, and 64.69 ± 4.63%; STAT1 were 41.06 ± 4.26%, 40.13 ± 3.43%, 44.98 ± 4.93%, and 48.78 ± 3.06%; FAS were 40.66 ± 4.94%, 42.59 ± 2.05%, 48.89 ± 3.98%, and 57.31 ± 3.84%; c-Fos were 71.67 ± 4.35%, 91.29 ± 4.6%, 89.83 ± 11.36%, and 63.79 ± 5.51%; NOS2 were 50.45 ± 4.47%, 48.34 ± 4.39%, 48.87 ± 7.47%, and 62.82 ± 5.26%; PTGS2 were 64.5 ± 7.38%, 53.73 ± 3.46%, 58.93 ± 6.62%, and 69.72 ± 5.44%; STAT3 were 73.34 ± 6.59%, 69.79 ± 6.06%, 103.01 ± 12.16%, and 77.04 ± 3.46%.

## 4. Discussion

These days, researchers have understandably started focusing on developing immuno-modulatory therapies and anti-inflammatory drugs that deal with inflammasome inhibition, pyroptosis suppression, toll-like receptor block, transcript factor deactivation, phosphoprotein dephosphorylation, mitogen-activated protein kinases deactivation, and so on. Moreover, studies on many herbal drugs and natural products have reported them to have anti-inflammatory effects without cytotoxicity [8,9,10].

For example, a study by Yoon et al. reported that *Scutellaria baicalensis* Georgi water extract inhibited LPS-induced production of NO, IL-3, IL-6, IL-10, IL-12p40, IL-17, IP-10, keratinocyte-derived chemokine (KC), and VEGF in mouse macrophages [11]. A study by Yuk et al. similarly reported that *Epimedium brevicornum* Maxim water extract inhibits LPS-induced production of NO, IL-3, IL-10, IL-12p40, IP-10, KC, VEGF, MCP-1, and GM-CSF in mouse macrophages [12]. These studies notably found that these herbal drugs did not present any cytotoxicity on macrophages because pyroptosis (the inflammatory programmed cell death, in which immune cells release pro-inflammatory cytokines, swell, burst, and die) is a distinct aspect of infection-induced intracellular inflammatory pathway in immune cells. Like *Scutellaria baicalensis* water extract and *Epimedium brevicornum* water extract, ASW (up to a concentration of 200 µg/mL) did not show any cytotoxic effect on macrophages in the present study. In addition to the anti-inflammatory effect of *Angelica sinensis* ethyl acetate extract on LPS, plus IFN-γ-induced RAW 264.7 [2]. Su et al. reported that ligustilide, a major compound of *Angelica sinensis* root, significantly suppressed NO, TNF-α, and prostaglandin E2 production from LPS-induced RAW 264.7 by deactivation of MAPK, NF-kappaB, and AP-1 [13]. Interestingly, our data represented that ASW inhibits excessive release of intracellular calcium as well as overproduction of NO and cytokines in LPS-induced RAW 264.7. However, it is a limitation that IL-10, a well-known anti-inflammatory cytokine, was decreased by ASW in the present assay. It is another shortcoming that this study could not evaluate the effect of ASW on mitogen-activated protein kinase cascade caused by LPS.

LPS-induced macrophages release many kinds of inflammatory mediators such as NO, interleukins, chemokines, growth factors, and so forth. Thus, LPS-stimulated mouse macrophage is a common model for examining the anti-inflammatory effect of natural products and herbal medicines. This is because LPS causes an endoplasmic reticulum (ER) stress with the overexpression of CHOP, which initiates inflammasome activation and subsequent macrophage pyroptosis [14].

Interestingly, although originally produced to fight against infectious pathogens, NO plays a role as an ER stress and brings about an unfolded protein response. In conjunction, as an effector of the unfolded protein response, CHOP amplifies the release of ER calcium store and activates STAT pathway [15,16,17]. Additionally, the JAK-STAT pathway has been already reported to play a critical role in the proinflammatory gene expression of RAW 264.7 [18].

Thus, it is meaningful to examine the effects of ASW on LPS-induced CHOP overexpression and pyroptosis signaling. In the present study, our experimental data showed that *Angelica sinensis* water extract inhibits the overexpression of CHOP, JAK2, STAT1, FAS, c-Fos, NOS2, and PTGS2 in LPS-induced macrophages.

We also found it pertinent to evaluate intracellular calcium. It is an important signaling molecule of ER stress and increases with CHOP overexpression by LPS induction.

Finally, we found that ASW restored the cell viability in LPS-induced RAW 264.7 at concentrations of up to 200 µg/mL. ASW showed notable anti-inflammatory effects at these concentrations. Data revealed that ASW exhibited IC_50_ = 954.3, 387.3, 191.7, 317.8, 1267.0, 347.0, 110.1, 573.6, 1171.0, 732.6, 980.8, 125.0, and 257.0 µg/mL for IL-6, TNF-α, MCP-1, RANTES, G-CSF, GM-CSF, VEGF, LIX, MIP-1α, MIP-1β, MIP-2, IL-10, and intracellular calcium, respectively. Additionally, ASW (25, 50, 100, and 200 µg/mL) inhibited the LPS-induced production of nitric oxide and the LPS-induced mRNA expression of CHOP, JAK2, STAT1, FAS, c-Fos, NOS2, and PTGS2 in RAW 264.7 significantly (*p* < 0.05). These suggest that ASW exerts anti-inflammatory effects in LPS-induced RAW 264.7 via NO-bursting/calcium-mediated JAK-STAT pathway. Further studies are needed to evaluate why ASW inhibited the production of IL-10 in LPS-induced RAW 264.7. Nevertheless, it is clear that ASW deserves to be intensely investigated for its usefulness in the treatment of hyper-inflammatory diseases with massive cytokine production.

## Figures and Tables

**Table 1:** Primers used for RT-PCR analysis.

Name ^1^	Forward Primer (5′-3′)	Reverse Primer (5′-3′)
STAT1	TGAGATGTCCCGGATAGTGG	CGCCAGAGAGAAATTCGTGT
STAT3	GTCTGCAGAGT TCAAGCACCT	TCCTCAGTCACGATCAAGGAG
CHOP	CGCTGTTTTCCCTTGCTG	TCCTCATACCAGGCTTCCA
JAK2	TTGGTTTTGAATTATGGTGTCTGT	TCCAAATTTTACAAATTCTTGAACC
FAS	CGCTGTTTTCCCTTGCTG	CCTTGAGTATGAACTCTTAACTGTGAG
c-Fos	AGAGCGGGAATGGTGAAGA	TCTTCCTCTTCAGGAGATAGCTG
NOS2	TGGAGGTTCTGGATGAGAGC	AATGTCCAGGAAGTAGGTGAGG
PTGS2	TCAAACAGTTTCTCTACAACAACTCC	ACATTTCTTCCCCCAGCAA
β-Actin	CTAAGGCCAACCGTGAAAAG	ACCAGAGGCATACAGGGACA

^1^ Primer’s names; signal transducers and activators of transcription (STAT) 1. STAT3, C/EBP homologous protein (CHOP), janus kinase 2 (JAK2), first apoptosis signal receptor (FAS), c-Fos, nitric oxide synthase 2 (NOS2), prostaglandin-ndoperoxide synthase 2 (PTGS2), β-Actin.

## Abbreviations

NO	Nitric oxide
ASW	*Angelica sinensis* root water extract
LPS	Lipopolysaccharide
JAK	Janus kinase
STAT	Signal transducers and activators of transcription
RE	rutin equivalents
IL	Interleukin
TNF	Tumor necrosis factor
G-CSF	Granulocyte colony-stimulating factor
GM-CSF	Granulocyte macrophage colony-stimulating factor
LIX	Lipopolysaccharide-induced CXC chemokine
MCP	Monocyte chemotactic activating factor
MIP	Macrophage inflammatory protein
RANTES	Regulated on activation, normal T cell expressed and secreted
VEGF	Vascular endothelial growth factor
IP-10	Interferon gamma-induced protein 10
LIF	Leukemia inhibitory factor
CHOP	C/EBP homologous protein
FAS	First apoptosis signal receptor
NOS2	Nitric oxide synthase 2
PTGS2	Prostaglandin-endoperoxide synthase 2
ER	Endoplasmic reticulum
KC	Keratinocyte-derived chemokine
